# Congenital panhypopituitarism unmasked by PHACE screening

**DOI:** 10.1016/j.jdcr.2023.07.003

**Published:** 2023-07-13

**Authors:** Paula Finnegan, Emma Tierney, Siobhan Rafferty, Orla Neylon, Muriel Sadlier

**Affiliations:** a Department of Dermatology, University Hospital Limerick, Dooradoyle, Co. Limerick, Ireland; b Department of Pediatric Endocrinology, University Hospital Limerick, Dooradoyle, Co. Limerick, Ireland

**Keywords:** endocrinopathy, haemangioma, hemangioma, hypopituitarism, panhypopituitarism, PHACE, PHACES, PHACE syndrome, segmental haemangioma

## Introduction

PHACES (OMIM-606519) is a congenital neurocutaneous syndrome describing the association of Posterior fossa brain malformations, segmental cervicofacial infantile Hemangiomas (IHs), Arterial anomalies, Cardiac defects, Eye anomalies, and ventral developmental defects, such as *S*ternal clefting or Supraumbilical raphe.^1^, ^2^, ^3^ Approximately 50% of patients with PHACE syndrome have structural brain anomalies.^4^ Congenital and acquired endocrinopathies are a well-recognized, although rare, disease association.^2^, ^3^, ^4^, ^5^, ^6^, ^7^, ^8^, ^9^ Some authors have even suggested that the “*E*” in PHACE could also represent endocrinopathy.^10^ Here, we present an infant with multifocal IHs and possible PHACE syndrome who was found to have congenital panhypopituitarism as a result of diagnostic screening for PHACE syndrome.

## Case report

A 7-week-old girl was referred to dermatology for assessment of multiple IH on her head and trunk. She was born at term following an uncomplicated pregnancy. Her birth weight was 2.8 kg. She had a history of neonatal hypotonia and hypoglycemia, which resolved after 2 days. Her heel prick test did not detect any abnormalities. There was no history of breathing or feeding difficulties. On review of systems, her parents reported infrequent stooling (every 2-3 days).

**Fig 1:**
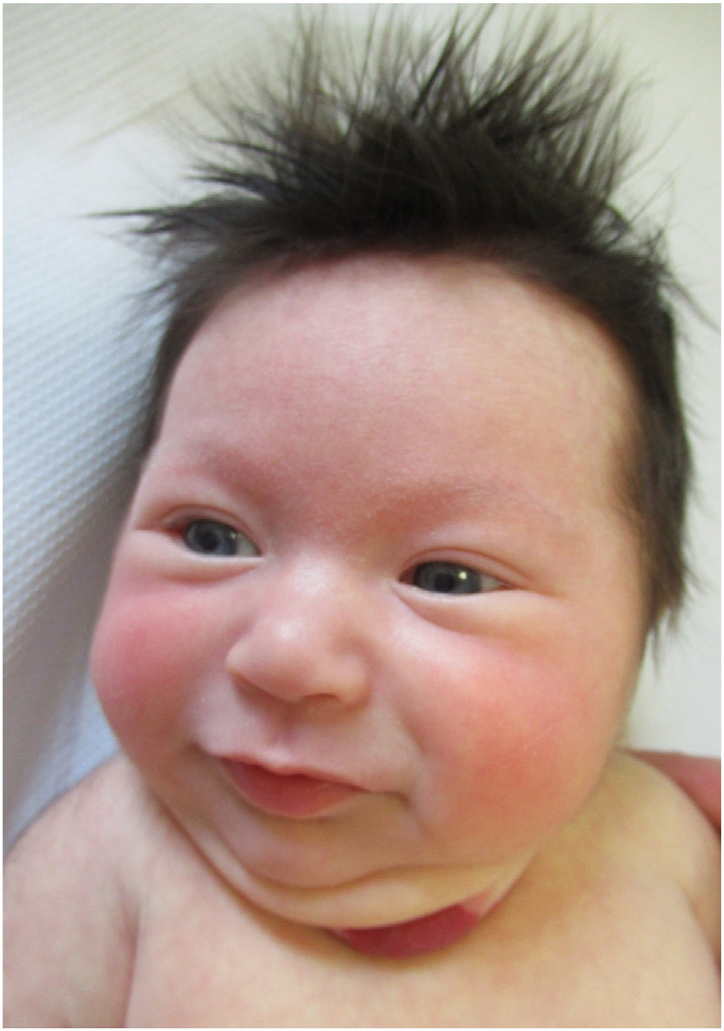
Infantile hemangiomas left side of the neck—anterior view.

**Fig 2:**
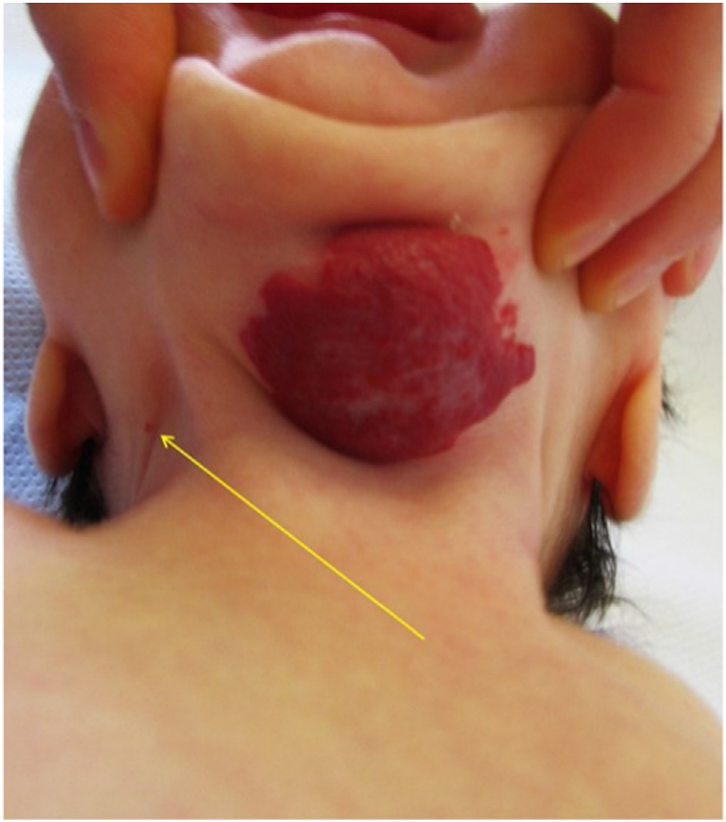
*Arrow* points to superficial vascular papule on lateral aspect of the neck.

On examination, she had a large superficial IH measuring 5 × 4 cm, which was slightly graying centrally on the midline of her neck. She had a small 1-mm superficial IHs lateral to this suggesting possible evolving segmental morphology (Figs 1 and 2). In addition, she had 3 superficial IH, each measuring between 1 and 2 cm, on the scalp and upper portion of the trunk. No sternal or midline defects were present. There were no signs of airway compromise. Because of the distribution and size of her neck IH, she underwent evaluation for PHACE.

**Fig 3:**
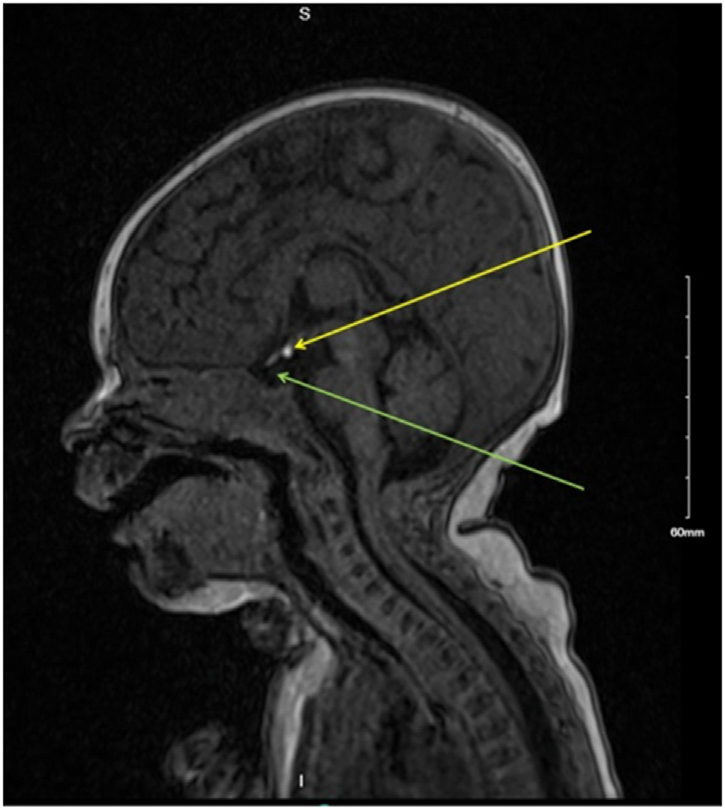
Magnetic resonance imaging of the brain (sagittal view); *yellow arrow* shows posterior pituitary gland; *green arrow* shows empty sella turcica.

Magnetic resonance imaging of the head and neck detected an ectopic pituitary gland. The pituitary stalk was not visualized. No vascular anomalies were present (Fig 3). The remaining PHACE workup, including airway evaluation was negative. Endocrinology assessment revealed extreme cortisol deficiency (<3 nmol/L [normal range 68-327]) and unrecordable adrenocorticotropic hormone levels (<0.7pmol/L [ref 2.2-13.3]). Gonadotrophins were undetectable (luteinizing hormone <1.0 U/L [ref 2.4-12.6], follicle-stimulating hormone <1.0 U/L [3.5-12.5]) when expected to be high, consistent with physiologic “mini-puberty” of infancy. She had a central pattern of hypothyroidism (thyroid stimulating hormone 1.81mU/L [ref 0.4-4.2], free T4 9 pmol/L [ref 10.5-22.8]). Serum prolactin level was elevated (882 mU/L [ref 102-496]). Insulin-like growth factor 1 (IGF-1) and IGF-binding protein 3 were normal. Screening for diabetes insipidus was negative, and no unmasking after starting other hormonal treatment was noted. This pattern is consistent with pituitary gland dysfunction or panhypopituitarism. She commenced hydrocortisone and levothyroxine replacement. She became noticeably more alert and active within days of starting treatment. Her IH was treated with propranolol 1 mg/kg every 8 hours with initial inpatient monitoring of blood glucose. She has had an excellent response to propranolol without any adverse events to date. Screening for developmental dysplasia of the hip was subsequently positive, necessitating several operative orthopedic interventions. She required hydrocortisone “stress dose” cover at anesthetic induction for each procedure. The infant is not currently on growth hormone replacement and is exhibiting steady growth velocity with IGF-1 and IGFBP-3 in low-normal ranges.

## Discussion

The pituitary gland is a central regulator of growth, metabolism, reproduction, and homeostasis. Panhypopituitarism occurs when there is absent or reduced production of multiple pituitary hormones.^11^ It is crucial to diagnose congenital hypopituitarism early as delay in hormone replacement therapy can have devastating consequences. Untreated hypopituitarism can result in premature death because of cardiovascular disease, cerebrovascular disease, or adrenal insufficiency.^11^ However, recognizing hypopituitarism in the neonatal period is difficult because symptoms are usually nonspecific. These include hypoglycemia, prolonged jaundice, poor weight gain, temperature dysregulation, electrolyte abnormalities, hemodynamic instability, or recurrent sepsis.

Congenital or acquired endocrinopathies occur more frequently in patients with PHACE syndrome. These can occur because of pituitary dysfunction or dysfunction elsewhere in the hormonal axis, such as the hypothalamus or target endocrine organs. Hypopituitarism is a minor diagnostic criterion for PHACE,^7^ although it is an extremely rare manifestation of the disease with just 4 reported cases (Table I).^2^^,^^3^^,^^5^^,^^6^^,^^9^ All patients were female, consistent with the known female predominance of PHACE. No other cases were diagnosed <1 year of age. One patient who became symptomatic at the age of 11 years developed secondary or acquired hypopituitarism because of compression of the adenohypophysis by an arachnoidal cyst located near the sella turcica.^3^ One patient did not have a segmental or facial IH; however, the patient had multiple typical structural brain and heart anomalies in association with multiple small cutaneous IH.^5^ Additionally, there is one other report of hypopituitarism in an infant with multifocal cutaneous IH. Investigations for failure to thrive demonstrated panhypopituitarism because of compression of the pituitary stalk by an internal hemangioma.^12^ Our patient did not fulfill the 2016 consensus criteria for PHACE, although she had possible PHACE (IH of the neck >5 cm and 2 minor criteria [midline brain anomaly and hypopituitarism]) in association with multifocal IH.

**Table I:** Summary of reported cases of PHACE with hypopituitarism

Authors	Gender	Age at diagnosis hypopituitarism	Hemangioma type	Other clinical features	Pituitary radiologic findings	Endocrine abnormalities	Other features PHACE
Goddard et al^2^ (2006) (also reported by Lasky et al^9^ [2004])	Female	13 mo	Large segmental facial haemangioma affecting bilateral frontal regions, left cheek, nose and upper lip and periorbital area (midline involvement)	Poor linear growth; motor and speech delay; oculomotor apraxia; hypotonia; increased weight gain	Absent pituitary bright spot; partially empty and deeply seated sella turcica	-Central hypothyroidism; growth hormone deficiency	**Neuro:** Thin corpus callosum, asymmetry of the lateral ventricles; ectatic left ICA; hypoplastic posterior arch of C1; cyst in posterior fossa **Cardiac:** VSD **Ocular:** coloboma-like iris defect
Denzer et al^3^ (2012)	Female	11 y	Large segmental facial haemangioma (midline involvement)	Short stature; reduced growth velocity;	Empty sella; dorsal displacement of the infundibulum by arachnoid cysts	Absolute GH deficiency; hypogonadotropic hypogonadism; central hypothyroidism; secondary adrenal insufficiency	**Neuro:** Right-sided cerebellar dysplasia, brainstem deformation & dislocation, & right carotid aplasia **Cardiac:** nil
Poindexter et al^5^ (2007)	Female	13 mo	Multiple (10) nonsegmental haemangiomas on trunk and neck	Low-set ears; bilateral talipes equinovarus; congenital left-sided facial palsy; right-sided sensorineural hearing loss	Ectopic neurohypophysis; thinned /absent infundibulum; reduced anterior pituitary volume	Hypothyroidism; adrenal insufficiency	**Neuro**: subependymal heteropia on lateral ventricles; persistent left trigeminal artery **Cardiac**: ASD; PDA
Merheb et al^6^ (2010)	Female	16 y	Right intraorbital haemangioma	Reduced growth velocity; primary amenorrhoea	Abnormal enhancement and thickening of the pituitary stalk & hypothalamus	Central hypothyroidism, GH deficiency, and hypogonadotropic hypogonadism	**Neuro**: Posterior fossa haemangioma; hypoplastic left internal carotid artery **Cardiac**: nil **Ocular**: mild exophthalmos

*ASD*, Atrial septal defect; *GH*, growth hormone; *ICA*, internal carotid artery; *PDA*, patent ductus arteriosus; *VSD*, ventricular septal defect.

Patients with noncervicofacial segmental IH and with nonsegmental large IH have been reported with structural abnormalities similar to those reported in PHACE. It has been hypothesized that PHACE occurs because of a developmental field defect with resultant clinical findings depending on the timing and location of this defect in the developing embryo. We believe that our patients large midline neck IH was related to their midline brain anomaly and consequent pituitary dysfunction; however, coincidental occurrence cannot be excluded.^11^

Structural pituitary gland abnormalities and endocrine dysfunction are underrecognized in patients with PHACE syndrome. Steiner et al^4^ retrospectively reviewed magnetic resonance imaging brain scans from 55 patients from the PHACE international registry and found pituitary anomalies in 18% of cases (*n* = 10). Full assessment, including careful history, physical examination, and pituitary panel, are required when pituitary pathologies are identified radiologically; however, we also believe that it is reasonable given the association between PHACE and endocrinopathies, and the potentially detrimental consequences of untreated endocrine disorders in infancy, to include a basic pituitary panel (thyroid function tests, morning cortisol, serum glucose, growth hormone, and gonadotropins levels) in patients with segmental or large IH being assessed for PHACE syndrome. Additionally, any patient with PHACE syndrome exhibiting poor growth, failure to thrive, or delayed puberty should have endocrine evaluation for possible acquired endocrinopathy.

In conclusion, we report a rare case of congenital panhypopituitarism detected by diagnostic screening for PHACE in an infant with minor nonspecific symptoms (transient neonatal hypotonia and hypoglycemia), multifocal IH, and possible PHACE. Early detection and treatment have potentially prevented life-threatening complications as a result of her multiple hormone deficiencies, particularly important given the coinciding developmental dysplasia of the hip pathology and well-described risk of adrenal crisis/severe hypoglycemia in an undiagnosed patient at or after anesthetic induction.

## Conflicts of interest

None disclosed.
